# Real-World Evidence from a Large German Outpatient Database for Oncology Research: A Narrative Review

**DOI:** 10.3390/cancers18111747

**Published:** 2026-05-27

**Authors:** Karel Kostev, Marcel Konrad, Andreas Krieg, Sarah Krieg

**Affiliations:** 1 Marburg University, University Clinic, 35043 Marburg, Germany; 2 Epidemiology, IQVIA, 60549 Frankfurt, Germany; 3 Health & Social, FOM University of Applied Sciences for Economics and Management, 60486 Frankfurt am Main, Germany; 4 Department of General and Visceral Surgery, Thoracic Surgery and Proctology, University Hospital Herford, Medical Campus OWL, Ruhr University Bochum, 32049 Herford, Germany; 5 University Clinic for People with Neurodevelopmental Disorders, Mara Hospital, Medical School and University Medical Center OWL, Bielefeld University, 33615 Bielefeld, Germany; sarah.krieg@mara.de

**Keywords:** cancer, oncology, database, epidemiology, incidence, comorbidity, sex-specific associations

## Abstract

**Background/Objectives:** The German IQVIA Disease Analyzer (DA) database, which contains data from more than 20 million patients in Germany, has become an important real-world data source for oncology research. This narrative review summarizes DA-based studies published between 2020 and 2025, focusing on methodological approaches, major thematic areas, and the contribution of outpatient routine data to cancer epidemiology. **Methods:** A structured PubMed search identified observational studies using the German DA database as the primary data source. Eligible publications examined cancer incidence, associated conditions, medication exposures, or survivorship outcomes in outpatient settings. Study designs, exposures, outcomes, and analytical strategies were synthesized qualitatively. **Results:** DA-based oncology studies predominantly used retrospective cohort and case–control designs. Research themes included associations between chronic inflammatory, metabolic, infectious, and cardiovascular conditions and subsequent cancer diagnoses; medication exposures and cancer incidence; and survivorship-related outcomes such as second primary cancers, fractures, and mental health disorders. Across cancer types, multimorbidity and long-term health trajectories emerged as consistent patterns. Strengths of the DA database include large unselected populations and longitudinal follow-up, while limitations relate to the absence of tumor-specific details, inpatient data, lifestyle factors, and mortality information. **Conclusions:** Outpatient routine data from the DA database provide valuable insights into cancer incidence, comorbidity patterns, and survivorship in real-world clinical practice. Although limited in oncological detail, DA-based studies complement registry and trial data and contribute to a broader understanding of cancer epidemiology in everyday healthcare settings.

## Simple Summary

Cancer is often diagnosed and managed in outpatient medical practices, yet most research relies on hospital data or cancer registries. The German Disease Analyzer database contains long-term information from general practitioners and specialists and therefore offers a unique view of how cancer develops and is treated in everyday clinical care. This review summarizes studies that used this database to explore how chronic diseases, infections, metabolic conditions, and medications are linked to later cancer diagnoses, as well as how cancer survivors experience long-term health problems. By bringing these findings together, the review shows how routine outpatient data can help researchers better understand cancer risks, comorbidities, and survivorship. These insights may support future research and improve real-world cancer prevention and care.

## 1. Introduction

Cancer remains one of the leading causes of morbidity and mortality worldwide, with global estimates documenting a continuous rise in incidence and substantial geographic variation [1,2,3]. Large international surveillance efforts, including CONCORD v3, have demonstrated improvements in survival for several malignancies while highlighting persistent inequalities across regions and socioeconomic groups [4,5]. Global burden analyses further show that cancer contributes significantly to disability adjusted life years and premature mortality, particularly in aging populations [6,7]. These trends underscore the need for robust epidemiological data to inform prevention, screening, and healthcare planning [8,9].

A substantial proportion of the global cancer burden is attributable to modifiable lifestyle and metabolic factors. Excess body fatness, diabetes, and obesity have been linked to increased risks of gastrointestinal, breast, and pancreatic cancers [10,11,12,13,14,15,16].

Comprehensive evaluations of preventable cancer burden in the United States estimate that a large share of incident cancers could be avoided through reductions in smoking, alcohol consumption, and other behavioral exposures [17,18]. More recent analyses have shown substantial state level variation in smoking attributable cancer mortality, reflecting differences in policy and prevention efforts [19]. Environmental and dietary exposures as well as gut microbiota also contribute to global cancer patterns, with notable increases in different cancer types [20,21,22,23,24].

Chronic inflammation, immune dysregulation, and infections represent additional key determinants of cancer risk. Foundational mechanistic work has established the role of inflammatory signaling pathways in tumor initiation and progression [25]. Autoimmune diseases and chronic inflammatory conditions have been associated with altered cancer susceptibility, reflecting long-standing immune activation [26]. Infection-related cancers remain a major global challenge, with Helicobacter pylori, hepatitis viruses, and Epstein–Barr virus accounting for a substantial proportion of cases worldwide [27]. Gastric cancer, in particular, continues to show strong links to infectious and inflammatory exposures [20].

Parallel to advances in prevention and early detection, the number of individuals living with and beyond cancer continues to grow. Survivorship research has documented increasing prevalence, long-term treatment-related complications, and elevated risks of second primary malignancies [28,29,30,31,32].

Psychiatric comorbidities such as depression and anxiety are common among cancer survivors and have important implications for quality of life and healthcare utilization [33,34]. Multimorbidity is now recognized as a defining feature of contemporary oncology, influencing diagnostic pathways, treatment decisions, and long-term outcomes [35,36,37,38].

Real-world data (RWD) sources have become increasingly important for understanding cancer epidemiology, multimorbidity, survivorship, and healthcare utilization in routine clinical settings. Electronic health records, administrative claims, and outpatient databases provide complementary insights to registries and clinical trials, particularly for elderly and multimorbid populations [39,40]. Methodological work has highlighted the strengths and limitations of using electronic health records for population-based research, including issues of data completeness, coding accuracy, and confounding [41].

Within this context, the German Disease Analyzer (DA) database, a large outpatient electronic medical record database maintained by IQVIA, represents a valuable platform for oncology research. By capturing longitudinal outpatient diagnoses, prescriptions, and healthcare utilization, the DA database enables the study of cancer incidence, comorbidity patterns, medication exposures, and survivorship-related outcomes in large, unselected populations. This narrative review synthesizes oncology research conducted with the DA database between 2020 and 2025 and situates these findings within the broader landscape of contemporary cancer epidemiology informed by the international literature cited above.

Although numerous oncology studies using the German Disease Analyzer database have been published in recent years, these investigations remain fragmented across different cancer entities, exposures, and methodological approaches. A specific gap in the existing literature is the absence of a dedicated synthesis of the methodological approaches employed in Disease Analyzer-based oncology research, including the study designs applied, their respective strengths and limitations, and the analytical strategies used across studies. To our knowledge, no previous review has comprehensively synthesized the recurring epidemiological patterns, methodological characteristics, interpretative limitations, and conceptual implications of Disease Analyzer-based oncology research in outpatient care. The present narrative review therefore aims to integrate findings from Disease Analyzer oncology studies published between 2020 and 2025 into a broader conceptual framework for outpatient real-world oncology research while critically discussing methodological approaches, strengths, limitations, and the interpretability of observed associations.

## 2. Review Type and Literature Search Strategy

This work is a narrative review summarizing oncology research conducted with the German IQVIA Disease Analyzer database. A structured literature search was performed in PubMed to identify eligible studies published between January 2020 and December 2025. PubMed was selected because the relevant Disease Analyzer oncology literature is primarily published in peer-reviewed biomedical and epidemiological journals indexed in this database, which was considered appropriate for the focused narrative scope of the review. Additional databases such as Scopus or Web of Science were not used because Disease Analyzer oncology publications are predominantly indexed in MEDLINE-covered journals, and pilot searches indicated that PubMed captured the relevant literature adequately without requiring broader multi-database coverage. Furthermore, PubMed/MEDLINE indexing itself serves as an indicator of journal quality and scientific rigor, as journals must meet stringent editorial, peer-review, and publication ethics criteria to be included, thereby ensuring that only publications meeting established scientific standards were considered in this review. Search terms included combinations of ‘Disease Analyzer’, ‘IQVIA’, ‘Germany’, ‘cancer’, ‘oncology’, ‘neoplasm’, ‘incidence’, and ‘risk’.

The literature selection process was conducted sequentially. First, titles and abstracts identified through the PubMed search were screened for relevance to outpatient oncology research using the German Disease Analyzer database. Second, full texts of potentially eligible articles were assessed for inclusion. Studies were included if they used the German Disease Analyzer database as the primary data source, investigated oncology-related outcomes, cancer incidence, survivorship, or cancer-associated exposures, were based on an observational study design, and were published in English. Articles not directly related to oncology research using the German Disease Analyzer database were excluded during screening. Reference lists of included studies were additionally screened to identify further relevant publications.

Study selection and eligibility assessment were performed by the authors, and disagreements regarding inclusion were resolved through discussion. Because the present manuscript was designed as a focused narrative review rather than a systematic review or meta-analysis, no formal PRISMA workflow or quantitative risk-of-bias scoring system was applied. Instead, methodological strengths, limitations, potential sources of bias, and interpretative constraints of the included studies were critically discussed qualitatively throughout the manuscript.

## 3. The Disease Analyzer Database as a Platform for Oncology Research

The IQVIA Disease Analyzer database comprises anonymized longitudinal data derived from the electronic medical records of German outpatient practices, including general practitioners, internists, gynecologists, gastroenterologists, and other non-hospital-based specialists. The information available includes ICD-10 diagnoses, ATC-coded prescriptions, consultation dates, and basic demographic characteristics such as age and sex. The database is designed to be representative of routine outpatient care in Germany with respect to physician specialty distribution and geographic coverage.

From an oncological perspective, the Disease Analyzer database offers a number of specific advantages. Many cancers are initially suspected, documented, or monitored in outpatient settings, particularly during early diagnostic work-up, long-term follow-up, and survivorship care. The DA database therefore supports the investigation of cancer incidence and associated conditions in large, unselected populations over extended observation periods. Unlike cancer registries, which focus on tumor-specific characteristics, the DA database reflects the broader clinical context in which cancer diagnoses are made, including multimorbidity and the utilization of healthcare over time.

Most oncology studies using DA data employ retrospective cohort or case–control designs. These designs allow for the evaluation of associations between pre-existing conditions, medication exposure, or infections and subsequent cancer diagnoses. Adjustment is commonly performed for age, sex, and consultation frequency, in recognition of the importance of healthcare utilization in outpatient data. Table 1 provides an overview of DA-based oncology studies published between 2020 and 2025.

## 4. Methodological Approaches in Disease Analyzer-Based Oncology Studies

Oncology studies using the Disease Analyzer database predominantly rely on retrospective observational designs tailored to routine outpatient data. Case–control studies are often used to evaluate the associations between pre-existing conditions or laboratory parameters and cancer incidence. In these analyses, patients with incident cancer diagnoses are matched to cancer-free controls based on factors such as age, sex, index year, and often also consultation frequency. This allows for the efficient assessment of prior exposures.

Retrospective cohort designs are commonly employed to examine cancer incidence following specific diagnoses, infections, or medication exposure. In such studies, cohort entry is usually defined by the first documentation of the exposure of interest, and patients are followed longitudinally until a cancer diagnosis, loss to follow-up, or end of data availability. Latency periods are often applied to reduce reverse causation, although their length varies between studies.

Cancer outcomes in DA-based studies are defined using ICD-10 codes documented in outpatient care. To increase diagnostic validity, many analyses require cancer diagnoses to be documented repeatedly across multiple visits. However, histological confirmation and staging information are not available in the DA database. As a result, DA-based oncology studies primarily focus on cancer incidence rather than disease progression or prognosis.

Adjustment strategies commonly include demographic variables and comorbid conditions documented prior to cancer diagnosis. Furthermore, consultation frequency is often included to account for differential healthcare utilization. Despite these measures, residual confounding remains an inherent limitation of observational studies based on routine data.

Retrospective cohort studies represent the preferred design for estimating cancer incidence, as they follow a defined population over time and allow direct measurement of cancer incidence rates following a specific exposure, diagnosis, or disease. Their key advantage is the ability to quantify how frequently cancer develops within an exposed cohort under routine-care conditions. Case–control studies, by contrast, are particularly suited to investigating multiple prior exposures, comorbidities, or treatments as factors associated with a cancer outcome. By comparing patients with and without cancer, these designs enable efficient assessment of a broad range of antecedent factors—including medication use, prior diagnoses, and therapeutic exposures—within the same analytical framework. Cross-sectional analyses are additionally useful for describing multimorbidity patterns and healthcare utilization in oncology populations.

At the same time, all included observational study designs are subject to important methodological limitations. Residual confounding, reverse causation, and differential healthcare utilization may influence observed associations despite statistical adjustment. Patients with chronic diseases often undergo more intensive diagnostic surveillance, potentially contributing to detection bias and selection effects. Furthermore, because diagnoses are based on outpatient coding practices, delayed documentation and diagnostic misclassification cannot be fully excluded. Consequently, Disease Analyzer-based oncology studies are particularly valuable for hypothesis generation and population-level epidemiological analyses, whereas causal interpretation remains limited.

## 5. Chronic Diseases and Cancer Incidence

### 5.1. Autoimmune and Inflammatory Diseases

This study category includes studies examining autoimmune, inflammatory, or chronic immune-mediated conditions as potential factors associated with subsequent cancer diagnoses.

Several DA-based studies have examined associations between autoimmune or inflammatory diseases and subsequent cancer diagnoses. Large case–control analyses have reported associations between autoimmune diseases and pancreatic cancer, with notable sex-specific differences [42]. These findings suggest that long-standing immune dysregulation may be reflected in altered cancer incidence patterns that are detectable in outpatient data.

Functional and inflammatory gastrointestinal disorders have also been investigated. Patients with irritable bowel syndrome have been shown to exhibit distinct cancer incidence patterns during follow-up [46]. In addition, analyses of comorbidity patterns in patients with colorectal cancer highlighted the high burden of chronic diseases documented prior to cancer diagnosis [45], emphasizing the role of multimorbidity in oncological populations.

### 5.2. Metabolic and Cardiovascular Conditions

This study category comprises studies investigating metabolic or cardiovascular disorders, laboratory markers, and related chronic conditions in relation to cancer incidence.

Metabolic dysregulation represents another key area of DA-based oncology research. A large case–control study found an association between elevated glycated hemoglobin levels and pancreatic cancer incidence, suggesting that impaired glucose metabolism could be detected years prior to a cancer diagnosis in routine-care settings [52]. Similarly, low HDL cholesterol levels were associated with gastrointestinal cancer diagnoses, with differences observed between men and women [44].

Cardiovascular conditions have also been studied. A retrospective cohort study [49] found that pulmonary hypertension was associated with an increased incidence of cancer diagnoses, illustrating the intersection between chronic cardiovascular disease and oncological outcomes in outpatient populations.

Although such associations are clinically relevant for hypothesis generation, they should be interpreted cautiously because retrospective outpatient data cannot establish causality and residual confounding related to multimorbidity and healthcare utilization may persist despite statistical adjustment.

### 5.3. Infections and Cancer Diagnoses

This study category includes studies evaluating bacterial or viral infections and their associations with subsequent cancer diagnoses in outpatient populations.

DA-based oncology studies have examined the associations between infectious diseases and subsequent cancer diagnoses. Helicobacter pylori infection and eradication therapy were evaluated in relation to cancer incidence, demonstrating heterogeneous associations depending on cancer type and treatment status [43]. These findings highlight the complexity of infection-related cancer epidemiology in routine care.

DA data have also been used to investigate viral infections in relation to cancer diagnoses. Infectious mononucleosis has been linked to a later cancer diagnosis in a cohort study, reflecting long-term oncological patterns following Epstein–Barr virus infection [50]. By contrast, herpes zoster was not associated with gastrointestinal cancer incidence [54] and also showed no association with breast cancer in a large cohort analysis [53]. These negative findings are important, as they provide real-world evidence that contradicts broad assumptions regarding the risk of infection-related cancer.

### 5.4. Medication Exposure and Cancer Incidence

This study category includes studies investigating the associations between medication exposure and subsequent cancer incidence or cancer-related outcomes.

Medication use is a recurring theme in DA-based oncology research. In one recent study, antibiotic therapy was associated with increased cancer incidence in a large outpatient cohort [48]. While causal mechanisms cannot be established, these findings raise questions regarding long-term microbiome alterations, immune modulation, and confounding by the underlying disease burden.

In the study of Stritzelberger et al., the use of anti-seizure medication in patients with epilepsy was not associated with an increased incidence of cancer, providing reassuring evidence from routine outpatient data [56].

Hormonal and phytotherapeutic treatments have also been studied using the DA database. Prescriptions of *Rheum rhaponticum* extract (ERr 731) were evaluated in relation to subsequent breast cancer diagnoses, contributing to the evidence base regarding non-hormonal therapies used in the management of menopausal symptoms [51].

### 5.5. Cancer Survivorship and Secondary Outcomes

This study category includes studies examining long-term outcomes among cancer survivors, including secondary malignancies, treatment-related complications, and mental health disorders.

Beyond incident cancer diagnoses, DA-based oncology research has addressed survivorship-related outcomes. Studies in women with breast cancer have examined the incidence of second primary cancers during long-term follow-up [55], demonstrating the feasibility of studying secondary oncological outcomes using routine-care data.

The DA database has also been used to investigate treatment-related complications. For example, a retrospective cohort study assessed fracture risk in postmenopausal women with breast cancer receiving chemotherapy or endocrine therapy, illustrating how DA data can be used to evaluate long-term adverse outcomes associated with cancer treatment [57].

Psychiatric outcomes in cancer patients represent another emerging area of research. A cohort study has described mental disorder diagnoses following colorectal cancer diagnosis, emphasizing the psychiatric burden that accompanies oncological disease in outpatient care [47]. These findings illustrate the intersection between oncology and mental health research within the same real-world data framework.

Beyond disease recurrence and secondary malignancies, cancer survivorship is increasingly characterized by long-term multimorbidity, psychological burden, and continued healthcare utilization. Outpatient routine data such as the Disease Analyzer database may therefore contribute to a better understanding of longitudinal care patterns among cancer survivors, including mental health disorders, chronic treatment-related complications, and the coordination of care across medical specialties. These aspects are particularly relevant in aging populations, where survivorship frequently overlaps with complex chronic disease management.

## 6. Cancer-Specific Considerations When Using Outpatient Routine Data

Several cancer-specific aspects must be considered when interpreting DA-based oncology studies. Cancer diagnoses are often initially established in hospital settings and subsequently documented in outpatient care. As a result, there may be delays between the actual diagnosis and its coding in DA, which can affect temporal analyses.

Furthermore, detection intensity varies across cancer types and patient groups. Sex-specific screening programs such as mammography or colorectal cancer screening influence the timing and likelihood of cancer diagnosis in outpatient data. Similarly, patients with chronic diseases may undergo more frequent diagnostic testing, potentially increasing cancer detection rates.

Differences between solid tumors and hematological malignancies must also be considered. Hematological cancers are more likely to be managed by specialized centers and may be underrepresented in outpatient data compared with solid tumors, which are commonly managed by general practitioners.

## 7. Cross-Cancer Synthesis of Findings from Disease Analyzer Studies

Several recurring patterns have emerged from DA-based oncology research across different cancer entities. One consistent observation is the association between chronic inflammatory, metabolic, or immune-mediated conditions and subsequent cancer diagnoses. These associations appear across different organ systems, suggesting that systemic disease burden plays an important role in cancer epidemiology observable in routine care.

Another recurring theme is the frequent coexistence of cancer with multimorbidity. DA-based studies consistently show that cancer diagnoses occur within complex longitudinal health trajectories rather than as isolated events. This emphasizes the importance of integrated care approaches for cancer patients. Across the reviewed studies, several recurring epidemiological patterns became apparent. Positive associations with cancer incidence were more consistently observed for chronic inflammatory, metabolic, autoimmune, and cardiovascular conditions than for transient infectious diseases. In contrast, several infection-related studies, particularly those investigating herpes zoster, showed no significant association with subsequent cancer diagnoses. These contrasting findings suggest that not all forms of immune activation or infection-related disease burden translate into measurable increases in cancer incidence in outpatient routine-care data. Differences between studies may partly reflect heterogeneity in study populations, exposure definitions, latency periods, healthcare utilization patterns, and diagnostic coding practices. Furthermore, studies investigating chronic systemic diseases may be more strongly influenced by multimorbidity burden and long-term diagnostic surveillance than studies evaluating acute infectious exposures. These observations emphasize the complexity of interpreting associations identified in retrospective outpatient oncology data.

Medication-related analyses reveal heterogeneous associations, highlighting the challenges in disentangling treatment effects from underlying disease severity and healthcare utilization. Negative findings such as the absence of associations between herpes zoster or anti-seizure medication and cancer incidence are equally important and contribute to a balanced evidence base.

Taken together, the reviewed studies suggest that outpatient real-world oncology data are particularly valuable for identifying longitudinal associations between multimorbidity, chronic inflammation, metabolic dysregulation, healthcare utilization patterns, and subsequent cancer diagnoses. However, many observed associations are likely influenced by complex interactions between underlying disease burden, diagnostic intensity, and residual confounding. Consequently, DA-based oncology studies are especially suitable for hypothesis generation and population-level epidemiological surveillance, whereas causal interpretation and tumor-specific biological inference require complementary data sources such as registries, molecular datasets, or prospective cohorts.

## 8. Strengths and Limitations of the Disease Analyzer Database in Oncology Research

Studies based on the IQVIA Disease Analyzer database offer several methodological strengths for oncology research. These include large sample sizes, nationwide coverage, and longitudinal follow-up under routine outpatient care conditions. The database facilitates the investigation of cancer incidence and associated conditions in unselected populations including elderly patients and individuals with multimorbidity, who are frequently underrepresented in clinical trials. Moreover, the consistent structure of DA data allows for the application of comparable study designs across different cancer entities, facilitating cross-study comparisons within the same data framework.

Overall, the reviewed studies were methodologically consistent in their use of large outpatient cohorts, longitudinal follow-up, and adjustment for demographic variables and healthcare utilization. However, most studies were retrospective observational analyses and are therefore inherently limited in their ability to establish causality. Furthermore, heterogeneity in study design, exposure definitions, latency periods, and confounder adjustment may influence comparability across studies. Despite these limitations, the consistency of several associations across independent analyses supports the relevance of outpatient routine data for hypothesis generation and real-world oncology research.

There are a number of important limitations that must be considered carefully when interpreting DA-based oncology findings. First, no detailed tumor-specific information is available. Cancer diagnoses are recorded using ICD-10 codes, but data on tumor stage, TNM classification, histological subtype, grading, and molecular or genetic markers are not captured. As a result, DA-based studies cannot differentiate between early and advanced disease, nor can they account for tumor biology. This limits their ability to assess prognosis, disease severity, or stage-specific associations.

Second, the database primarily reflects outpatient care. Information from hospital settings, including inpatient diagnostics, surgical procedures, chemotherapy administered during hospitalization, radiotherapy, and acute complications, is not fully captured. In addition, data from specialized oncologist practices are limited or absent, depending on the cancer entity and healthcare pathway. Consequently, DA-based oncology studies mainly describe cancers as documented in general practice or non-oncological specialist care, which may result in the underrepresentation of aspects of specialized cancer management.

Third, the Disease Analyzer database contains no mortality data. This precludes analyses of cancer-specific survival, overall survival, and cause-specific mortality. As a result, DA-based studies are generally restricted to incident cancer diagnoses and intermediate outcomes rather than hard oncological endpoints. Competing risks, such as death from non-cancer causes, cannot be accounted for directly.

Fourth, important behavioral and lifestyle factors are not recorded. Information on smoking status, alcohol consumption, diet, physical activity, body composition beyond basic measures, and occupational or environmental exposures is lacking. These factors are highly relevant in oncology and may confound the associations between chronic diseases, medication use, and cancer incidence. Their absence limits the ability to adjust for lifestyle-related confounding factors and may contribute to residual bias.

Fifth, there are no data available on psychosocial factors and functional status. Variables such as physical mobility, frailty, social support, and quality of life, which are increasingly recognized as being relevant to cancer epidemiology and survivorship research, cannot be assessed using DA data.

Finally, as with all routine-care databases, diagnostic accuracy depends on physician coding practices. Misclassification, delayed documentation, or underdiagnosis may occur, particularly for cancers diagnosed in hospital settings and subsequently recorded in outpatient care. Although many studies employ strategies such as requiring repeated diagnostic documentation to improve validity, a certain degree of misclassification cannot be ruled out.

Taken together, these limitations suggest that Disease Analyzer-based oncology studies should be interpreted as providing evidence of associations that are observable in routine outpatient care, rather than detailed oncological characterization or causal inference.

Table 2 summarizes major methodological limitations of the Disease Analyzer database and commonly applied analytical strategies used to mitigate these constraints in oncology research.

DA-based research is therefore best viewed as complementary to cancer registries, hospital-based datasets, and clinical trials, contributing population-level insights into cancer epidemiology under real-world conditions.

## 9. Comparison with Other Real-World Oncology Data Sources

Unlike cancer registries, the Disease Analyzer database does not cover tumor-specific details such as TNM classification, staging, and histology but offers a broader clinical context and longitudinal outpatient follow-up. Although claims databases provide comprehensive billing information, they often lack clinical nuance. The DA database occupies an intermediate position, capturing real-world diagnostic and prescribing behavior while enabling population-based analyses.

Clinical trials and prospective cohorts offer high levels of internal validity but limited generalizability. DA-based studies complement these data by reflecting populations receiving routine care, including elderly and multimorbid patients.

Compared with Surveillance, Epidemiology, and End Results (SEER)-linked datasets [58,59,60], which combine cancer registry information with detailed survival and treatment-related data, the Disease Analyzer database provides less tumor-specific information but offers broader insight into longitudinal outpatient care, multimorbidity, and prescribing behavior in routine clinical practice. In contrast to imaging-focused resources such as The Cancer Imaging Archive (TCIA) [61], which primarily support radiological and computational oncology research, the Disease Analyzer database is designed for epidemiological and healthcare utilization studies based on routine outpatient documentation. These complementary characteristics illustrate the distinct role of the Disease Analyzer database within the spectrum of real-world oncology data sources.

The generalizability of findings derived from the German outpatient setting may vary across healthcare systems with different referral structures, insurance models, and roles of primary care physicians in cancer management. Nevertheless, the methodological principles underlying real-world data research, including longitudinal routine-care analyses and multimorbidity assessment, are broadly applicable internationally. Depending on national healthcare infrastructures, real-world oncology research may rely more strongly on cancer registries, claims databases, hospital-based datasets, or integrated electronic health record systems.

## 10. Conclusions

Oncology research using the German IQVIA Disease Analyzer database has provided valuable real-world insights into cancer incidence, comorbidity patterns, medication exposure, and survivorship-related outcomes in routine outpatient care. While it must be acknowledged that the database is subject to certain limitations regarding tumor-specific detail and lifestyle data, DA-based studies are an important addition to registry- and trial-based evidence in contemporary cancer epidemiology.

Future oncology research using DA data could be enhanced by linking it with cancer registries or hospital datasets to improve diagnostic resolution and outcome assessment. There is also substantial potential for interdisciplinary research at the intersection of oncology, psychiatry, and internal medicine, particularly in the areas of survivorship and multimorbidity research.

Future developments in real-world oncology research may include linkage approaches combining outpatient routine-care databases with molecular, genomic, or biomarker-based datasets. Such integration could improve the understanding of cancer heterogeneity, individualized risk profiles, and treatment-related outcomes under real-world conditions. In this context, the Disease Analyzer database may serve as a valuable complementary resource for population-based translational oncology research.

Beyond their epidemiological relevance, findings derived from Disease Analyzer data may also support healthcare policy and outpatient cancer care planning. Real-world evidence on multimorbidity, survivorship, mental health burden, and healthcare utilization may help identify vulnerable patient populations and inform prevention strategies, long-term follow-up concepts, and interdisciplinary care models in routine oncology practice.

## Figures and Tables

**Table 1:** Overview of Disease Analyzer studies in the field of oncology.

First Author (Year)	Cancer Entity	Main Exposure/Focus	Study Design	Setting	Number of Patients Included	Follow-Up in Years
Loosen (2025) [42]	Pancreatic	Autoimmune diseases	Case–control	Primary care	32,640	–
Loosen (2024) [43]	Multiple	Helicobacter pylori eradication	Retrospective cohort	Primary care	50,634	5
Loosen (2022) [44]	GI cancers	Dyslipidemia, LDL-. and HDL cholesterol	Case–control	Primary care	61,936	–
Loosen (2022) [45]	Colorectal	Comorbidity patterns	Cross-sectional	Primary care	35,648	–
Loosen (2021) [46]	Multiple	Irritable bowel syndrome	Retrospective cohort	Primary care	43,462	10
Roderburg (2024) [47]	Colorectal	Mental disorders after diagnosis	Retrospective cohort	Primary care	93,714	5
Roderburg (2023) [48]	Multiple	Antibiotic use	Retrospective cohort	Primary care	223,656	10
Roderburg (2022) [49]	Multiple	Pulmonary hypertension	Retrospective cohort	Primary care	22,218	10
Roderburg (2022) [50]	Multiple	Infectious mononucleosis	Retrospective cohort	Primary care	25,080	10
Heger (2025) [51]	Breast	ERr 731 prescription	Retrospective cohort	Gynecological care	22,744	10
Grewe (2024) [52]	Pancreatic	HbA1c levels	Case–control	Primary care	10,092	–
Krishnan (2024) [53]	Breast	Herpes zoster	Retrospective cohort	Primary care	128,510	10
Leyh (2023) [54]	GI cancers	Herpes zoster	Retrospective cohort	Primary care	206,246	10
Nikolov (2022) [55]	Breast	Second primary cancers	Retrospective cohort	Primary care	42,248	10
Stritzelberger (2021) [56]	All cancers	Anti-seizure medication	Retrospective cohort	Primary care	6304	10
Stumpf (2020) [57]	Breast	Fractures after cancer therapy	Retrospective cohort	Primary care	8230	5

**Table 2:** Major limitations of the Disease Analyzer database and commonly applied mitigation strategies in oncology research.

Limitation of DA Database	Potential Impact	Common Mitigation Strategy in DA-Based Studies
No TNM/staging information	Limited tumor characterization	Use of ICD-10 codes for lymph node and distant metastases
No histology/genomic markers	No molecular subtype analyses	Focus on population-level epidemiology
Limited mortality data	No cancer-specific survival analyses	Use of follow-up duration and longitudinal observation
Missing smoking/alcohol/lifestyle data	Residual confounding	Use of surrogate diagnoses such as COPD, tobacco dependence, alcohol-related disorders
No inpatient treatment data	Underrepresentation of advanced oncology care	Focus on outpatient incidence and survivorship research
Retrospective observational design	No causal inference	Matching, multivariable adjustment, sensitivity analyses, target trial emulations
Differential healthcare utilization	Detection bias	Adjustment for consultation frequency
Coding variability	Misclassification risk	Requirement for repeated diagnostic documentation

## Data Availability

No new data were created or analyzed in this study.

## References

[1] Sung H., Ferlay J., Siegel R.L., Laversanne M., Soerjomataram I., Jemal A., Bray F. (2021). Global cancer statistics 2020: GLOBOCAN estimates of incidence and mortality worldwide for 36 cancers in 185 countries. CA Cancer J. Clin..

[2] Siegel R.L., Giaquinto A.N., Jemal A. (2024). Cancer statistics, 2024. CA Cancer J. Clin..

[3] Bray F., Ferlay J., Soerjomataram I., Siegel R.L., Torre L.A., Jemal A. (2018). Global cancer statistics 2018: GLOBOCAN estimates of incidence and mortality worldwide for 36 cancers in 185 countries. CA Cancer J. Clin..

[4] Yoshida I., Di Carlo V., Matz M., Yamashita N., Teramoto N., Oki I., Shibata A., Nakata K., Saito M.K., Matsuzaka M. (2026). Trends in survival for adult patients with hematopoietic malignancies in Japan, 2000–2014 (CONCORD-3). Jpn. J. Clin. Oncol..

[5] Allemani C., Matsuda T., Di Carlo V., Harewood R., Matz M., Nikšić M., Bonaventure A., Valkov M., Johnson C.J., Estève J. (2018). Global surveillance of trends in cancer survival 2000–14 (CONCORD-3): Analysis of individual records for 37,513,025 patients diagnosed with one of 18 cancers from 322 population-based registries in 71 countries. Lancet.

[6] (2019). Global Burden of Disease Cancer Collaboration. Global, Regional, and National Cancer Incidence, Mortality, Years of Life Lost, Years Lived with Disability, and Disability-Adjusted Life-Years for 29 Cancer Groups, 1990 to 2017: A Systematic Analysis for the Global Burden of Disease Study. JAMA Oncol..

[7] Vineis P., Wild C.P. (2014). Global cancer patterns: Causes and prevention. Lancet.

[8] Arnold M., Sierra M.S., Laversanne M., Soerjomataram I., Jemal A., Bray F. (2017). Global patterns and trends in colorectal cancer incidence and mortality. Gut.

[9] Li C., Lei S., Ding L., Xu Y., Wu X., Wang H., Zhang Z., Gao T., Zhang Y., Li L. (2023). Global burden and trends of lung cancer incidence and mortality. Chin. Med. J..

[10] Lauby-Secretan B., Scoccianti C., Loomis D., Grosse Y., Bianchini F., Straif K., International Agency for Research on Cancer Handbook Working Group (2016). Body fatness and cancer—Viewpoint of the IARC Working Group. N. Engl. J. Med..

[11] Menini S., Iacobini C., Vitale M., Pesce C., Pugliese G. (2021). Diabetes and pancreatic cancer—A dangerous liaison relying on carbonyl stress. Cancers.

[12] Sánchez-Maldonado J.M., Collado R., Cabrera-Serrano A.J., Ter Horst R., Gálvez-Montosa F., Robles-Fernández I., Arenas-Rodríguez V., Cano-Gutiérrez B., Bakker O., Bravo-Fernández M.I. (2022). Type 2 diabetes-related variants influence the risk of developing prostate cancer: A population-based case-control study and meta-analysis. Cancers.

[13] Gallo M. (2024). Diabetes and cancer: Diabetes and Cancer: The Perfect Storm and a PRICE to Pay. Cancers.

[14] Moore S.C., Ryan P.J. (2026). Obesity and cancer: Methodological frontiers for mechanistic discoveries. PLoS Med..

[15] Del Console P., Catalano S., Győrffy B. (2026). Overweight and obesity increase breast cancer risk in postmenopausal women: A meta-analysis of 38 observational studies. Geroscience.

[16] Counts B.R., Bonetto A., Chan H.L., Au E.D., Jiang Y., Couch M.E., Guttridge D.C., Ostrowski M.C., Koniaris L.G., Zimmers T.A. (2026). High-fat diet and obesity each increase tumor cell proliferation and muscle wasting in experimental cancer cachexia. Am. J. Physiol. Cell Physiol..

[17] Islami F., Goding Sauer A., Miller K.D., Siegel R.L., Fedewa S.A., Jacobs E.J., McCullough M.L., Patel A.V., Ma J., Soerjomataram I. (2018). Proportion and number of cancer cases and deaths attributable to potentially modifiable risk factors in the United States. CA Cancer J. Clin..

[18] Zhang F.F., Cudhea F., Shan Z., Michaud D.S., Imamura F., Eom H., Ruan M., Rehm C.D., Liu J., Du M. (2019). Preventable cancer burden associated with poor diet in the United States. JNCI Cancer Spectr..

[19] Islami F., Bandi P., Sahar L., Ma J., Drope J., Jemal A. (2021). Cancer deaths attributable to cigarette smoking in 152 U.S. metropolitan or micropolitan statistical areas, 2013–2017. Cancer Causes Control.

[20] Arnold M., Abnet C.C., Neale R.E., Vignat J., Giovannucci E.L., McGlynn K.A., Bray F. (2020). Global burden of 5 major types of gastrointestinal cancer. Gastroenterology.

[21] Ratajczak-Pawłowska A.E., Jezierska K., Szymczak-Tomczak A., Zawada A., Rychter A.M., Skoracka K., Dobrowolska A., Krela-Kaźmierczak I. (2025). Lifestyle and breast cancer: Prevention and treatment support. Cancers.

[22] Menegassi B., Vinciguerra M. (2025). Ultraprocessed food and risk of cancer: Mechanistic pathways and public health implications. Cancers.

[23] Motevalli M., Stanford F.C. (2025). Personalized lifestyle interventions for prevention and treatment of obesity-related cancers: A call to action. Cancers.

[24] Shi W., Song B., Xia S., Jia D. (2026). The role of gut microbiota in obesity-related cancers. Semin. Cancer Biol..

[25] Grivennikov S.I., Greten F.R., Karin M. (2010). Immunity, inflammation, and cancer. Cell.

[26] Simon T.A., Thompson A., Gandhi K.K., Hochberg M.C., Suissa S. (2015). Incidence of malignancy in adult patients with rheumatoid arthritis: A meta-analysis. Arthritis Res. Ther..

[27] de Martel C., Georges D., Bray F., Ferlay J., Clifford G.M. (2020). Global burden of cancer attributable to infections in 2018: A worldwide incidence analysis. Lancet Glob. Health.

[28] Nekhlyudov L., Campbell G.B., Schmitz K.H., Brooks G.A., Kumar A.J., Ganz P.A., Von Ah D. (2022). Cancer-related impairments and functional limitations among long-term cancer survivors: Gaps and opportunities for clinical practice. Cancer.

[29] Jung J.M., Kim D.H., Kim Y.J., Moon I.J., Lee W.J., Chang S.E., Lee M.W., Won C.H. (2025). Risk of second primary malignancies among survivors of cutaneous melanoma: A nationwide population-based study in the Republic of Korea. Sci. Rep..

[30] Hemade A., Hallit S. (2024). Risk of second primary cancers in nodal non-Hodgkin lymphoma patients by primary lymph node location: A retrospective cohort population-based study. Ann. Med. Surg..

[31] Hu J.N., Zhuang X., Peng J., Rong H., Gao X., Xu N., Chen Q.J., Ma K. (2026). Second primary malignancy risk after thymic epithelial tumors: A Surveillance, Epidemiology, and End Results analysis integrating multiple primary-standardized incidence ratio and competing-risk models. J. Thorac. Dis..

[32] Buchler T., Ambrozova M., Majek O., Dianova T., Klika P., Dusek L. (2025). Risk of second primary malignancies after adjuvant chemotherapy for colon cancer. Cancer.

[33] Niedzwiedz C.L., Knifton L., Robb K.A., Katikireddi S.V., Smith D.J. (2019). Depression and anxiety among people living with and beyond cancer: A growing clinical and research priority. BMC Cancer.

[34] Bach A., Knauer K., Graf J., Schäffeler N., Stengel A. (2022). Psychiatric comorbidities in cancer survivors across tumor subtypes: A systematic review. World J. Psychiatry.

[35] Sarfati D., Koczwara B., Jackson C. (2016). The impact of comorbidity on cancer and its treatment. CA Cancer J. Clin..

[36] Abbad-Gomez D., Domingo L., Comas M., Santiá P., Jansana A., Poblador B., Sanz T., Del Cura I., Ibañez B., Padilla M. (2024). Effect of comorbidity and multimorbidity on adherence to follow-up recommendations among long-term breast cancer survivors. Maturitas.

[37] O’Hanlon S., Baxter M., Liposits G. (2026). Practical aspects of managing multimorbidity in older adults with cancer. Curr. Opin. Support Palliat. Care.

[38] Venchiarutti R.L., Dhillon H., Ee C., Hart N.H., Jefford M., Koczwara B. (2026). Priorities for multimorbidity management and research in cancer: A Delphi study of Australian cancer survivors, clinicians, and researchers. J. Cancer Surviv..

[39] Booth C.M., Karim S., Mackillop W.J. (2019). Real-world data: Towards achieving the achievable in cancer care. Nat. Rev. Clin. Oncol..

[40] Burns L., Le Roux N., Kalesnik-Orszulak R., Christian J., Dudinak J., Rockhold F., Khozin S., O’Donnell J. (2023). Real-world evidence for regulatory decision-making: Updated guidance from around the world. Front. Med..

[41] Casey J.A., Schwartz B.S., Stewart W.F., Adler N.E. (2016). Using Electronic Health Records for Population Health Research: A Review of Methods and Applications. Annu. Rev. Public Health.

[42] Loosen S.H., Hansen F.J., Luedde T., Roderburg C., Kostev K. (2025). A sex-dependent association between the history of autoimmune disease and the development of pancreatic cancer: A case–control study of 32,640 patients. Front. Oncol..

[43] Loosen S.H., Mertens A., Klein I., Leyh C., Krieg S., Kandler J., Luedde T., Roderburg C., Kostev K. (2024). Association between *Helicobacter pylori* and its eradication and the development of cancer. BMJ Open Gastroenterol..

[44] Loosen S.H., Kostev K., Luedde M., Luedde T., Roderburg C. (2022). Low blood levels of high-density lipoprotein (HDL) cholesterol are positively associated with cancer. J. Cancer Res. Clin. Oncol..

[45] Loosen S.H., Schöler D., Labuhn S., Mertens A., Jördens M.S., Luedde M., Kostev K., Luedde T., Roderburg C. (2022). The spectrum of co-diagnoses in patients with colorectal cancer: A retrospective cohort study of 17,824 outpatients in Germany. Cancers.

[46] Loosen S.H., Jördens M.S., Luedde M., Modest D.P., Labuhn S., Luedde T., Kostev K., Roderburg C. (2021). Incidence of cancer in patients with irritable bowel syndrome. J. Clin. Med..

[47] Roderburg C., Loosen S.H., Leyh C., Krieg A., Krieg S., Jördens M., Luedde T., Kostev K. (2024). Temporal trends in mental disorder rates among patients with colorectal cancer: A comprehensive analysis. J. Clin. Med..

[48] Roderburg C., Loosen S.H., Joerdens M.S., Demir M., Luedde T., Kostev K. (2023). Antibiotic therapy is associated with an increased incidence of cancer. J. Cancer Res. Clin. Oncol..

[49] Roderburg C., Loosen S.H., Hippe H.J., Luedde T., Kostev K., Luedde M. (2022). Pulmonary hypertension is associated with an increased incidence of cancer diagnoses. Pulm. Circ..

[50] Roderburg C., Krieg S., Krieg A., Luedde T., Kostev K., Loosen S.H. (2022). The association between infectious mononucleosis and cancer: A cohort study of 24,190 outpatients in Germany. Cancers.

[51] Heger P.W., Hotz D., Kalder M., Kostev K. (2025). Association between *Extract Rheum rhaponticum* 731 (ERr 731) prescription and subsequent breast cancer. Breast Cancer Res. Treat..

[52] Grewe S., Jördens M.S., Roderburg C., Leyh C., Labuhn S., Luedde T., Krieg S., Krieg A., Loosen S.H., Kostev K. (2024). Elevated HbA1c levels are associated with a risk of pancreatic cancer: A case-control study. J. Clin. Med..

[53] Krishnan V.D., Gremke N., Hajek A., Kostev K., Kalder M. (2024). No significant association between herpes zoster and breast cancer: A German outpatient study with over 120,000 participants. Clin. Pract..

[54] Leyh C., Roderburg C., Luedde T., Loosen S.H., Kostev K. (2023). Herpes zoster is not associated with subsequent gastrointestinal cancer: Data from over 200,000 outpatients in Germany. J. Cancer Res. Clin. Oncol..

[55] Nikolov I., Kostev K., Kalder M. (2022). Incidence of other cancer diagnoses in women with breast cancer: A retrospective cohort study with 42,248 women. Breast Cancer Res. Treat..

[56] Stritzelberger J., Lang J.D., Mueller T.M., Reindl C., Westermayer V., Kostev K., Hamer H.M. (2021). Anti-seizure medication is not associated with an increased risk to develop cancer in epilepsy patients. J. Neurol..

[57] Stumpf U., Kostev K., Siebenbürger G., Böcker W., Hadji P. (2020). Influence of chemotherapy and endocrine treatment on fractures in postmenopausal women with breast cancer—A retrospective cohort study. J. Bone Oncol..

[58] O’Rorke M.A., McDowell B.D., Xu T., DeCook R.R., Gryzlak B.M., Rudzianski N.J., Serrano K.C., Wehrheim A.M., Grewal U.S., Chandrasekharan C. (2026). Assessing patient characteristics in neuroendocrine tumor research: A comparison of the NET-PRO study to SEER population-based data. Endocrine.

[59] Chen Q., Shao Y., Liu C., He Y., Liu H. (2026). Log odds of positive lymph nodes predicts survival in metaplastic breast cancer based on SEER analysis. Discov. Oncol..

[60] Xi K., Wu Y., Sun X., Du C., Wang F., Liu J., Yin Y., Wang Y., Liu J., Li G. (2026). Prognostic significance of lymph node-related indices and a novel nomogram for rectal cancer patients with synchronous liver metastases after preoperative chemoradiotherapy: A population-based study. Transl. Cancer Res..

[61] Clark K., Vendt B., Smith K., Freymann J., Kirby J., Koppel P., Moore S., Phillips S., Maffitt D., Pringle M. (2013). The Cancer Imaging Archive (TCIA): Maintaining and operating a public information repository. J. Digit. Imaging.

